# Assessing Rotation-Invariant Feature Classification for Automated Wildebeest Population Counts

**DOI:** 10.1371/journal.pone.0156342

**Published:** 2016-05-26

**Authors:** Colin J. Torney, Andrew P. Dobson, Felix Borner, David J. Lloyd-Jones, David Moyer, Honori T. Maliti, Machoke Mwita, Howard Fredrick, Markus Borner, J. Grant C. Hopcraft

**Affiliations:** 1 Centre for Mathematics and the Environment, University of Exeter, Penryn Campus, Penryn, Cornwall, United Kingdom; 2 Department of Ecology & Evolutionary Biology, Princeton University, Princeton, New Jersey, United States of America; 3 Frankfurt Zoological Society, Serengeti National Park, Seronera, Tanzania; 4 Independent researcher, P.O. Box 1272, Iringa, Tanzania; 5 Integrated Research Center, The Field Museum of Natural History, 1400 S. Lake Shore Drive, Chicago, United States of America; 6 Tanzania Wildlife Research Institute, P.O.Box 661, Arusha, Tanzania; 7 Tanzania Conservation Resource Centre, Arusha, Tanzania; 8 Institute of Biodiversity, Animal Health and Comparative Medicine; Boyd Orr Centre for Population and Ecosystem Health, University of Glasgow, Glasgow, United Kingdom; Universidade de Aveiro, PORTUGAL

## Abstract

Accurate and on-demand animal population counts are the holy grail for wildlife conservation organizations throughout the world because they enable fast and responsive adaptive management policies. While the collection of image data from camera traps, satellites, and manned or unmanned aircraft has advanced significantly, the detection and identification of animals within images remains a major bottleneck since counting is primarily conducted by dedicated enumerators or citizen scientists. Recent developments in the field of computer vision suggest a potential resolution to this issue through the use of rotation-invariant object descriptors combined with machine learning algorithms. Here we implement an algorithm to detect and count wildebeest from aerial images collected in the Serengeti National Park in 2009 as part of the biennial wildebeest count. We find that the per image error rates are greater than, but comparable to, two separate human counts. For the total count, the algorithm is more accurate than both manual counts, suggesting that human counters have a tendency to systematically over or under count images. While the accuracy of the algorithm is not yet at an acceptable level for fully automatic counts, our results show this method is a promising avenue for further research and we highlight specific areas where future research should focus in order to develop fast and accurate enumeration of aerial count data. If combined with a bespoke image collection protocol, this approach may yield a fully automated wildebeest count in the near future.

## Introduction

Aerial surveys, in which the abundance of a population is estimated by flying transects over its habitat and counting the number of animals within a given sampling strip, are an essential tool for assessing wildlife population numbers [1, 2]. Many species are monitored in this way, including birds [3–5], land mammals [6–9], and aquatic fauna [10, 11]. While in-air counts are still used (i.e. animals are enumerated as they are encountered by observers), a common approach, especially with aggregated species living in high densities, is to employ aerial photography and then later count animals within images. The second stage of this process is frequently a labour-intensive procedure [12] that requires highly-skilled counters.

Automating the process of counting animals in images would therefore relieve a significant burden on governmental and non-governmental conservation organizations. Repeated measures of the population size over time allows managers to not only develop accurate estimates of the true population size, but it also enables the estimation of critical parameters about the population such as rates of recruitment, mortality, immigration and emmigration. These diagnostic parameters provide an early warning indicator of a population’s health and are core metrics of any adaptive management system. Therefore, increasing the accuracy and the processing speed of a population count enables managers to access critical data and implement preemptive management strategies at an early stage, rather than waiting months for the results to be counted. Furthermore, an automated counting system could increase the frequency between consecutive population counts and thereby increase the temporal resolution of trends.

Achieving automated animal counts has been the subject of extensive research [13–17]. This research forms part of the rapidly evolving field of machine learning and computer vision [18]. Applications of these techniques are diverse and recent advances include the accurate detection of faces [19], facial expressions [20], pedestrians [21], and handwritten text [22]. In the context of ecology and conservation, machine learning has been deployed to classify species based on vocalisations [23], to identify behavioural states [24], and to track and identify moving animals [25]. However the most significant application has been in the automation of animal census methods, either through direct enumeration of animals [13, 14], or through computer-aided mark recapture methods based on automatic identification of individuals [26–28].

In this work, we evaluate the performance of a recently proposed method for the classification of objects [29]. The method is based on the popular histogram of oriented gradients technique [21] but has the distinct advantage of extracting only rotationally invariant features; thus making it suitable for aerial survey images in which animals may be oriented in any direction. We apply the method to the complete set of survey images taken during the 2009 Serengeti National Park wildebeest count. The wildebeest count is performed every 2 to 3 years and involves flying transects at an altitude of 350–400ft above ground. The aircraft travels at a speed of 120–180kph (subject to wind speed and direction), with images taken every 10 seconds from a camera mounted through the floor of the aircraft [30]. The result is approximately 2000 images that take 3 weeks for a single individual to count. To test whether the method proposed by [29] is able to automate the counting of wildebeest we implemented the algorithm, automatically counted the 2009 images, then compared the performance of the method to the manual totals. By testing the method on this dataset we are able to comprehensively evaluate its performance in an applied setting on a task of genuine ecological importance.

## Materials and Methods

### Rotation-invariant image classifiers

For completeness we include here a brief description of the method employed to extract invariant features from images. This is based on [29] and we refer interested parties to that work for a more complete description of their method.

The histogram of oriented gradients (HOG) technique [21] is a popular method that uses the distribution of gradients within regions of images to classify objects. Liu et al. [29] modified this approach so that instead of using a discrete grid, HOG cells are treated as continuous functions that may be approximated using Fourier series. The advantage of this approach is that the extracted features of the image are constant even if the underlying object within the image rotates.

As in [29], to process an image we first construct a matrix of gradients in complex form from the grayscale image *I* using a finite-difference scheme

$$\begin{array}{c} G=\left(\frac{\partial }{\partial x}+i\frac{\partial }{\partial y}\right)I. \end{array}$$ (1)

Hence, each element of G denotes the gradient at the corresponding image pixel in the form Δ*x* + *i*Δ*y*. In polar coordinates this may be written as *re*^*iθ*^. If we consider each element of G as an individual cell [21] then the distribution of gradients is effectively a Dirac delta function, centred at *θ*,

$$\begin{array}{c} H(\phi )=r\delta (\theta -\phi ). \end{array}$$ (2)

Performing a Fourier series expansion of a Dirac delta function leads to

$$\begin{array}{c} H\left(\phi \right)=\sum_{m=-\infty }^{\infty }F_{m}e^{-2\pi im\phi }=\sum_{m=-\infty }^{\infty }re^{2\pi im(\theta -\phi )} \end{array}$$ (3)

Truncating this series at some maximum mode means we are left with a sequence of complex valued coefficients which represent the Fourier transform of the image gradient. The gradient at each pixel is therefore encoded by a sequence of Fourier coefficients and the full transformed image is stored in a 3-dimensional complex array, representing *x* and *y* coordinates and the modes of the Fourier transform. We denote the 2-d array of mode *m* coefficients as $F_{m}$.

**Fig 1:**
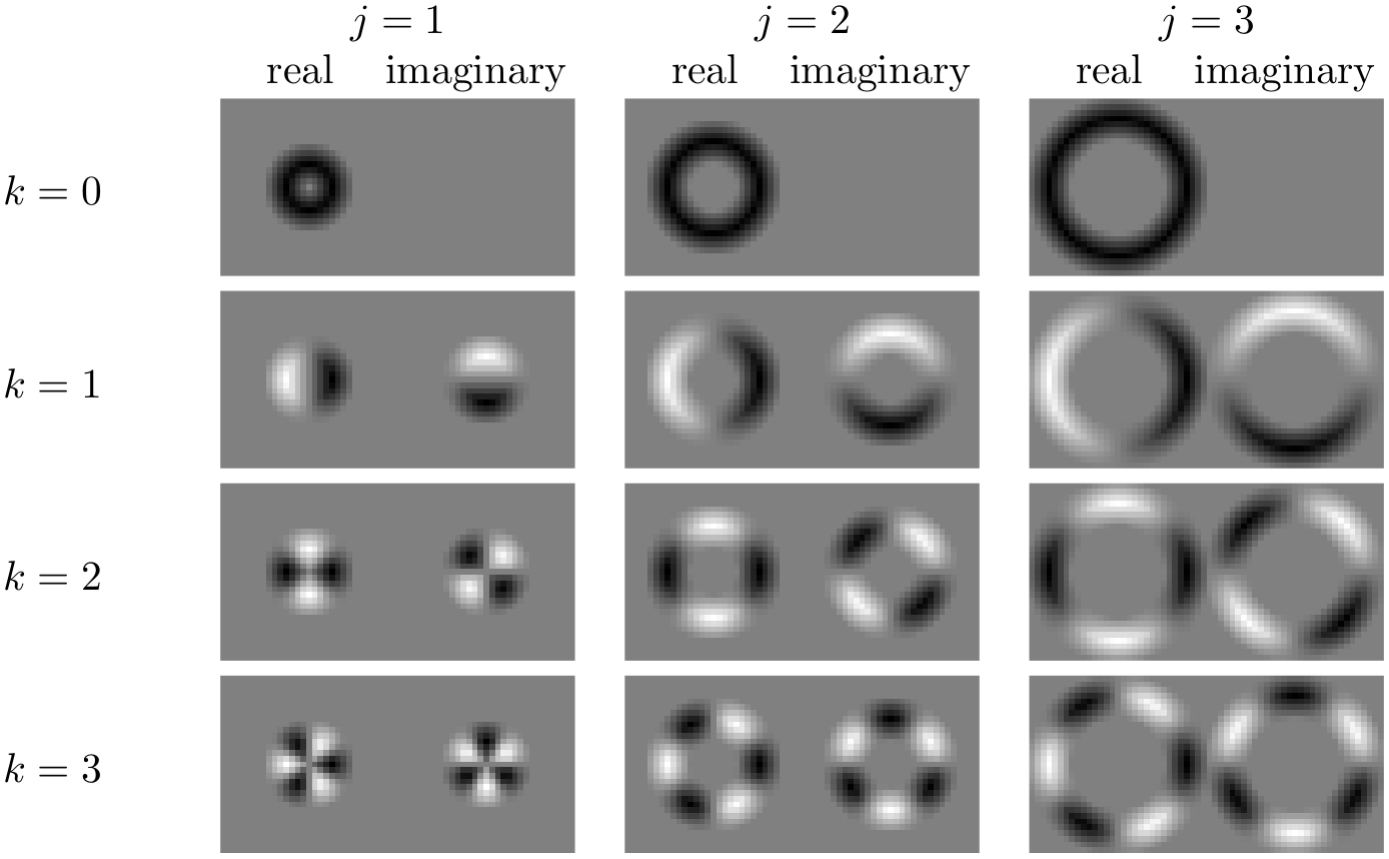
The basis functions, *U*_*j*, *k*_, for performing convolutions are constructed from Fourier modes on concentric circles. The parameter *j* determines the radial distance from the centre of the object, while *k* is the wavenumber. The images show the real and imaginary part of the basis function.

Next we introduce the Fourier basis functions *U*_*j*, *k*_ shown in Fig 1. By performing a convolution between a basis function *U*_*j*, *k*_ and a Fourier gradient field $F_{m}$ we obtain a Fourier HOG feature

$$\begin{array}{c} X_{k,m}=U_{j,k}*F_{m} \end{array}$$ (4)

which encodes information about the image gradients in the region covered by the basis function. These radially symmetric basis functions act in a manner equivalent to the cells of the original HOG method.

If the original image *I* is rotated then each of the complex valued features *X*_*k*, *m*_ will also be altered, i.e. in this form they are not invariant to rotations. However due to the shift property of Fourier analysis, rotations of the original image can be mapped to multiplications of the Fourier coefficients. A rotation of the original image by an angle *α* will result in the movement of pixels to another location and a rotation in the orientation of the gradients. These two effects can be mapped to the HOG feature by firstly rotating the Fourier transform of the gradient field $F_{m}$ by *α*, and secondly by rotating the basis functions *U*_*j*, *k*_ by −*α*.

If *X*_*k*, *m*_ is the original HOG feature, and *X*′_*k*, *m*_ is the corresponding feature calculated after the image has been rotated by *α*, then

$$\begin{array}{ll} {X^{\prime }}_{k,m} & =e^{-i\alpha k}U_{j,k}*e^{i\alpha m}F_{m}\\ & =e^{i\alpha \left(m-k\right)}\left(U_{j,k}*F_{m}\right)\\ & =e^{i\alpha \left(m-k\right)}X_{k,m} \end{array}$$ (5)

From this equation we can see that if *m* = *k* then image rotations have no impact on the descriptor and it is rotation invariant. Also, by taking the product of two descriptors, *X*_*k*1, *m*1_ and *X*_*k*2, *m*2_, a composite descriptor is formed,

$$\begin{array}{c} X_{k1,m1}X_{k2,m2}=e^{i\alpha (m1-k1+m2-k2)}\left(U_{j,k1}*F_{m1}\right)\left(U_{j,k2}*F_{m2}\right) \end{array}$$ (6)

Again we note that the composite descriptor remains constant for all angles of rotation angle *α* if (*m*1 − *k*1 + *m*2 − *k*2) = 0. We may therefore construct rotational invariant features of the image from the features defined by Eq 4, by firstly using features for which *m* − *k* = 0, and secondly by taking the product of any two features for which (*m*1 − *k*1 + *m*2 − *k*2) = 0.

### Implementation

The 2009 wildebeest count resulted in 2,018 images taken with a Nikon D2X 35mm camera shooting 4288x2848 pixel JPG images. Three separate counts of the aerial images were performed. Firstly, two independent counts were performed simultaneously by two different individuals. A third count was then performed by three individuals for images where there was a discrepancy between initial counts. This final count is taken to be the correct count for our comparison metrics. To evaluate the Fourier HOG method, the full 2009 image set was counted using machine learning software. The adaboost algorithm [31] was employed with a decision tree underlying classifier. Training images were drawn from 100 images taken from the 2012 survey.

The code was written in Python 2.7 (www.python.org) using OpenCV [32] for image operations and the sci-kit learn package [33] for classification. The classification code was parallelized using PyCUDA [34] and the code was run on an NVIDIA GeForce GT 630 graphics card. All code is based on open source libraries and is available here https://github.com/ctorney/wildCount

An iterative process was employed to train the classifier based on the 2012 images. First a set of sample images was generated from the 2012 image set by manually locating wildebeest. Next the classifier was trained on this small training data set and several further images were automatically counted. The results from this count were manually checked and corrected then used to create a larger training data set of 3000 positive samples and 3000 negative samples.

The trained classifier was then applied to the 2009 image set. Images were converted to grayscale then they were scanned for regions above a threshold level of local contrast. Regions that were uniform were discarded. Next each pixel that was in a non-uniform region was taken to be the centre of an object to be classified and rotation-invariant Fourier HOG features were extracted. Each pixel was then classified either as a wildebeest or not, then contiguous blocks of pixels were grouped and counted as a single individual.

## Results

**Fig 2:**
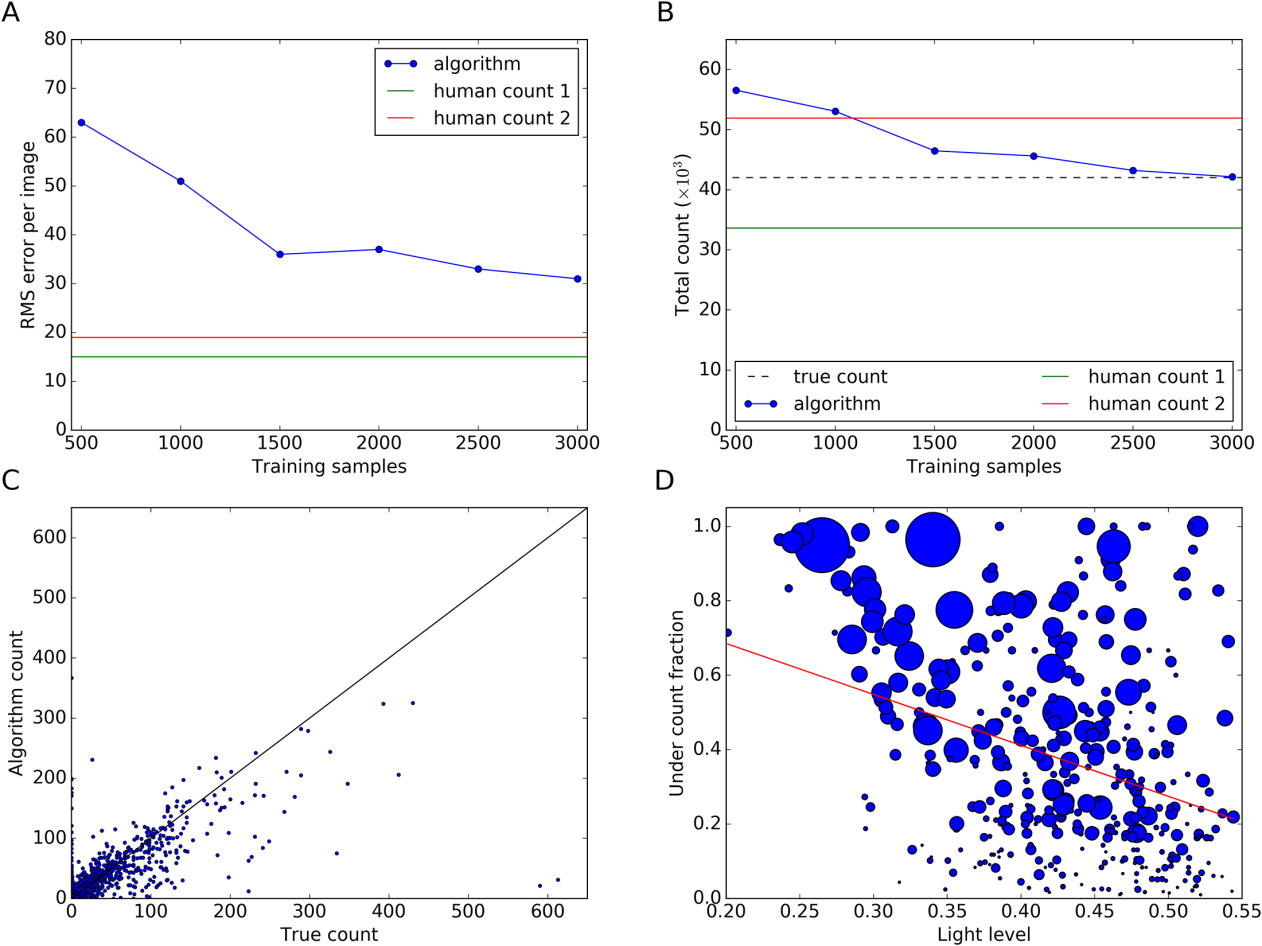
Comparing the performance of automated and manual counters. (A) Root mean square error of counts. The correct count for each image is assumed to be the third and final count. Average per image error is shown for the algorithm (blue line) as a function of the number of training samples from the 2012 survey that were used. For comparison, per image error is shown for each of the first pass human counts (red, green lines). (B) Total wildebeest counted within the image set. The final count is shown by the dashed line. The algorithm (blue line) outperforms both human counters in attaining a closer estimate to the true value. This is because the algorithm exhibits no systematic tendency to over or under count. It should be noted that 3000 was the maximum number of training samples available, and it is plausible that the automated total count will drop below the true count before it asymptotes. (C) Individual image errors. The black line is the *y* = *x* line for reference. While average per image errors are comparable between automated and human counters, the algorithm makes large errors in a small subset of images. Images that contain many false negatives tend to be darker than the training samples, while false positives occur when there is a lot of structure in the landscape. (D) A comparison of image light levels and under counting. A linear regression shows a significant negative relationship between image light level (average of value component of HSV image) and the amount of under counting (*β*_1_ = −1.37, *R*^2^ = 0.12). The under count fraction is calculated as $1-\frac{\text{algorithm count}}{\text{true count}}$ and images for which algorithm count > true count are excluded. Point sizes are proportional to the absolute value of the under count of wildebeest in the image.

To assess the accuracy of the method, total wildebeest counts are compared to the multiple counts performed by human counters. When using 3000 training examples for each class (positive or negative) we find good agreement between the automated totals and the manual counts as shown in Table 1. In Fig 2 performance of the algorithm is assessed against the final manual count and the two prior counts are shown for comparison. We note that while the automated total is more accurate than either initial counts, the root mean square error per image is greater. This metric, calculated as $\sqrt{\frac{1}{N}\sum D_{i}^{2}}$ where *D*_*i*_ is the difference in the count for image *i* and the summation is taken over all *N* images, reveals that the greater overall accuracy is due to the lack of any systematic bias in the machine learning algorithm. The mean error (defined as $\frac{1}{N}\sum D_{i}$) in Table 1 shows that each first pass manual count had either a positive or negative bias, whereas the algorithm displayed little systematic bias and was therefore able to obtain a more accurate total count, despite displaying a greater RMS error.

**Table 1:** Comparison of counts between manual and automated methods.

	Total	Mean (per image)	Coefficient of variation	RMS error	Mean error
First manual count	33644	16.67	2.61	15.64	-4.14
Second manual count	51918	25.73	2.21	19.75	4.91
Final manual count	42007	20.82	2.32	-	-
Automated count	42147	20.89	1.83	31.70	0.07

To measure the precision and recall of the method, 100 images were randomly selected and the number of true and false positives, and true and false negatives, were recorded. These results are shown in Table 2.

**Table 2:** Confusion matrix. As the accuracy based on the total count does not indicate precision or recall, performance metrics were recorded for a random subset of 100 images. Negative totals are based on the number of non-overlapping regions within each image that are approximately equal in area to a single wildebeest. From these results: precision $=\frac{TP}{TP+FP}=74.15\%$, recall $=\frac{TP}{TP+FN}=85.83\%$.

		Algorithm	
		Positive	Negative
Actual	Positive	1423	235
	Negative	496	1.2m

## Discussion

The advance in new technologies such as earth-orbiting satellites [14] or unmanned aerial vehicles [9], has led to a rapid increase in high-resolution, easily accessible image data. To keep pace with this progress, computational tools are required to automate image processing and ensure that these vast amounts of data are transformed into useful information. One area where modern computer vision techniques have the potential to significantly improve current practices is in the automated detection of animals within aerial count images. We have implemented a recent object classification method [29] which uses rotation-invariant features and is therefore suitable for use with these types of images.

**Fig 3:**
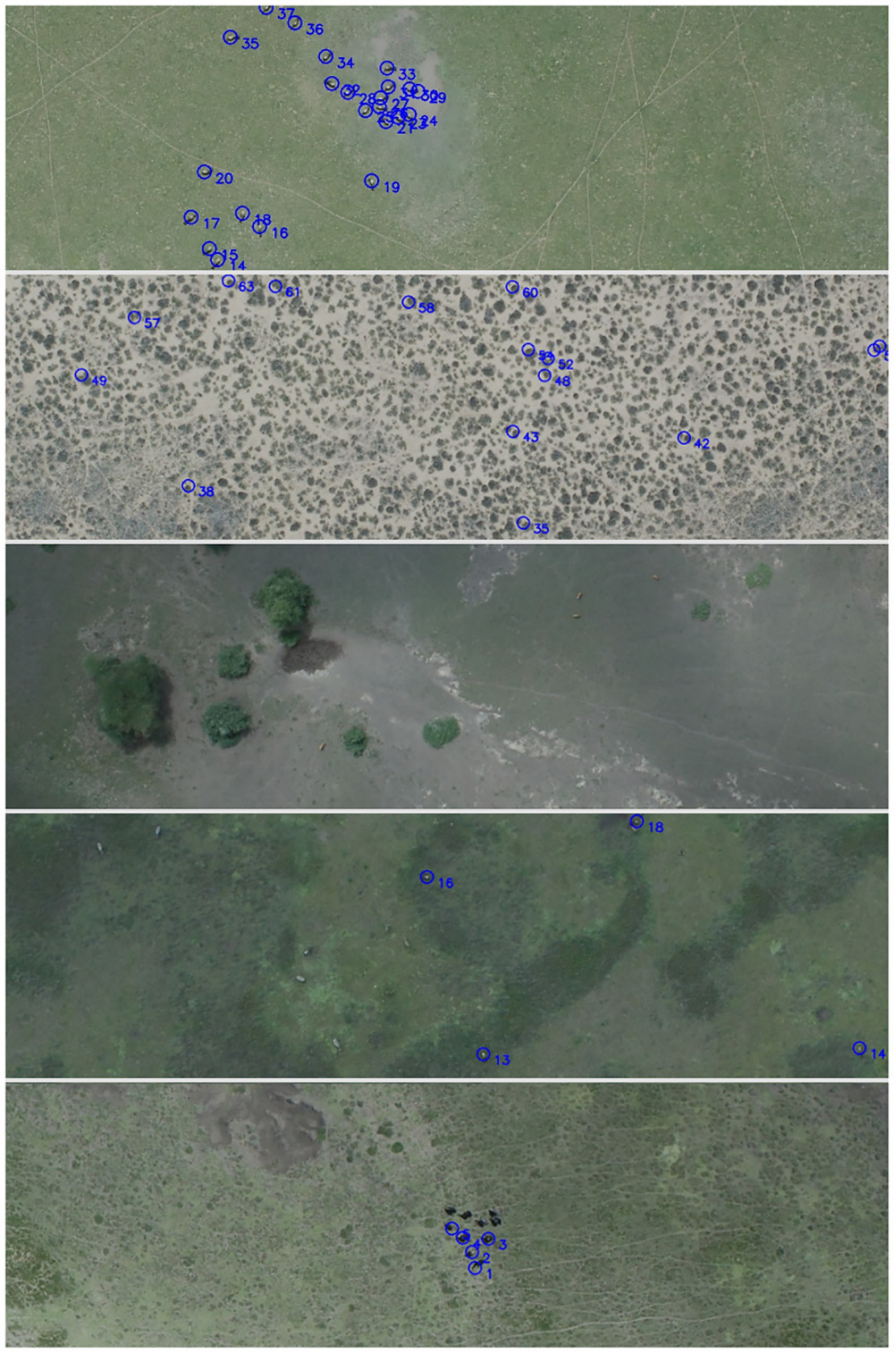
Example images. From top: Correctly detected wildebeest; Pattern and structure in the landscape frequently lead to false positives; The method is able to distinguish between different species; Species such as zebra, that have distinct body shapes are frequently not identified as wildebeest; The ability to distinguish between species is dependent on sufficient training examples, here the algorithm has misidentified a flock of juvenile ostrich as wildebeest.

By testing the method against multiple manual counts we find that its performance is comparable to a first-pass human count. The algorithm has a greater per image error rate, but is overall more accurate than two individual human counters. This is due to a lack of any systematic bias in errors, with landscape features leading to high rates of false positives, and low light conditions leading to false negatives (see Fig 3 for example images). Currently the algorithm is unlikely to outperform multiple human counts, either by trained professionals or through a citizen science approach that averages many counts by non-specialist individuals (such as the snap-shot Serengeti project operated through the zooniverse platform). However a combination of automated and manual counting would represent an ideal application of the method in its current form, either as a first-pass count or as a method to assess the performance of citizen scientists.

A significant promising aspect of the method is that it appears able to identify and differentiate between animal species. Although we were unable to quantify the performance of the algorithm in this regard with the current data set, these preliminary results show that common species such as zebra may be distinguished from wildebeest by the algorithm. In future we intend to further test this performance with training and testing data sets of multiple species.

A further avenue for future research will involve acquiring 3-dimensional information about the scene. As the features used by the classification method are based on the shape of the object, the error rate will be greatly reduced by obtaining the 3-dimensional structure of the object. This could be achieved through range imaging techniques, such as structure from motion [35] or LIDAR [36]. An alternative approach to increase accuracy would be to include a near-infrared thermal band that could differentiate between endothermic animals and the background. Both thermal and 3-d information could be used in combination with image gradients to enhance the accuracy of the method.

## Supporting Information

S1 File Count data. Human and automated counts for each image for 500, 1000, 1500, 2000, 2500, and 3000 training samples.
(ZIP)
